# Therapeutic approach to IgG4-related disease: A systematic review

**DOI:** 10.1097/MD.0000000000004002

**Published:** 2016-07-01

**Authors:** Pilar Brito-Zerón, Belchin Kostov, Xavier Bosch, Nihan Acar-Denizli, Manuel Ramos-Casals, John H. Stone

**Affiliations:** a Autoimmune Diseases Unit, Department of Medicine, Hospital CIMA-Sanitas; b Sjögren Syndrome Research Group (AGAUR), Laboratory of Autoimmune Diseases Josep Font, IDIBAPS-CELLEX; c Department of Autoimmune Diseases, ICMiD, Hospital Clínic; d Consorci d’Atenció Primària de Salut Barcelona Esquerre (CAPS-BE); e Transverse Group for Research in Primary Care, Institut d’Investigacions Biomèdiques August Pi i Sunyer (IDIBAPS); f Department of Internal Medicine, ICMiD, Hospital Clínic, Barcelona, Spain; g Department of Statistics, Faculty of Science and Letters, Mimar Sinan Fine Arts University, Istanbul, Turkey; h Department of Medicine, University of Barcelona, Barcelona, Spain; i Harvard Medical School and Department of Medicine (Division of Rheumatology, Allergy, and Immunology), Massachusetts General Hospital, Boston, MA.

**Keywords:** autoimmune pancreatitis, glucocorticoids, IgG4, IgG4-related disease, rituximab

## Abstract

To review the reported evidence on the therapeutic management of IgG4-related disease (IgG4-RD) in clinical practice.

A systematic search of the literature was conducted. The primary outcome measured was the rate of efficacy of first-line therapeutic approaches. Secondary outcomes measured included the rate of disease relapse, the outcome of untreated patients, the rate of patients without drug therapy at the end of follow-up, the rate of side effects, and mortality. The MOOSE, AHRQ, STROBE, and GRACE recommendations/statements were followed.

The results of the systematic search strategy yielded 62 studies that included a total of 3034 patients. Complete information about first-line therapeutic regimens was detailed in 1952 patients, including glucocorticoid-based regimens in 1437 (74%), drug-free regimens in 213 (11%), and other therapies in 38 (2%). No therapy (wait and see management) was reported in 264 (13%) patients. The efficacy of monotherapy with glucocorticoids was specified in 1220 patients, of whom 97% had a therapeutic response. Relapses, however, were reported in 464/1395 (33%) patients despite typically short follow-up periods. Therapeutic efficacy was reported in 219/231 (95%) of relapses treated with glucocorticoids, 56/69 (81%) of those treated with azathioprine, 16/22 (72%) of those treated with other immunosuppressive agents, and in the 9 cases treated with rituximab (100%). In 14 studies, the authors detailed the outcome of 159/246 patients with wait-and-see management; spontaneous improvement or resolution was reported in 68 (43%) cases. Wide heterogeneity was observed with respect to the first-line therapeutic approaches used for the different organ-specific disease subsets, including significant differences in the mean dose of glucocorticoids used.

Nearly 70% of reported IgG4-RD patients are treated with oral glucocorticoids in monotherapy. However, the therapeutic management is heavily influenced by geographical, epidemiological, and clinical factors, especially with respect to the predominant organ affected. The frequency of glucocorticoid failure to induce sustained remissions both during and after treatment and the assessment of glucocorticoid toxicity in IgG4-RD require further study.

## Abstract 2

Supplemental Digital Content is available in the text

## 1 Introduction

IgG4-related disease (IgG4-RD) is an immune-mediated systemic disease first described in Japan at the beginning of this century.^[1]^ The most-common clinical presentation is the development of a mass lesion or unexplained enlargement of one or more organs. Although IgG4-RD usually presents with a subacute onset, the disease leads to progressive organ failure and even death in some patients. Multiorgan disease is easier to identify at diagnosis but may evolve metachronously, with 1 organ at a time being added over months to years.^[1]^ The organs most frequently involved are the pancreas and biliary tract, the salivary and lachrymal glands, the kidneys, the thyroid gland, the lungs, and the aorta.^[2]^ Diagnosis relies on the coexistence of various clinical, laboratory, radiological, and histopathological findings, although none is pathognomonic in itself.^[3]^

The optimum therapeutic management of IgG4-RD has not yet been established.^[4]^ In spite of an explosion in observational studies in the last decade, no randomized controlled studies on the treatment of IgG4-RD are yet available. Therefore, in the absence of high-quality scientific evidence, a systematic approach using meta-analyses of observational studies is one of the few ways to assess the degree of efficacy of the therapeutic options currently used in clinical practice. This information may be useful for the design of future prospective/controlled therapeutic trials in IgG4-RD patients.

The objective of this review was to summarize reported evidence on the therapeutic management of IgG4-RD in clinical practice with the aim of providing physicians with the best available therapeutic evidence, tailored when possible to the possible clinical scenarios with which IgG4-RD present.

## 2 Methods

### 2.1 Data sources and searches

A systematic search of the literature was conducted on MEDLINE and EMBASE and using the OVID interface to search for evidence-based medicine reviews in the Cochrane Databases of systematic reviews and controlled trials. We also searched for unpublished trials using ClinicalTrials.gov (last day of access to the databases, October 31, 2014). Due to the lack of specific Medical Subject Headings for IgG4-RD, a text-word search was conducted using the free text “IgG4-related disease” as the currently most-accepted term.^[1]^ No restrictions were placed on language or type of publication. We manually searched reference lists of selected articles for relevant citations that our searches missed. We followed the AMSTAR recommendations on data sources and searches.^[5]^

### 2.2 Study selection

Study selection was made by independent review. Two independent reviewers (MRC and XB) examined abstracts retrieved by the literature search for potentially eligible articles. Studies marked for possible inclusion by either reviewer underwent dual, independent full-text review. Differences between reviewers were resolved by consensus. Criteria for inclusion were the following: inclusion of at least 5 patients, enrollment of adults (aged ≥18 years), diagnosis of IgG4-RD according to current classification criteria sets,^[6]^ and availability of data on at least one of the following outcomes: therapeutic efficacy; disease outcomes (relapses, maintenance drug therapy, and/or death); and/or adverse drug effects.

### 2.3 Data extraction and quality assessment

A data extraction form was developed by PBZ prior to manuscript review to gather relevant data from each article. All data extractions were reviewed for completeness and accuracy by PBZ, XB, and MRC. Study design was classified according to the STROBE recommendations for observational studies (case–control, cross-sectional, and cohort studies).^[7]^ Variables collected for each study selected included the mean age of the cohort, gender frequencies, country, inclusion criteria, first-line therapeutic approaches, drug therapies (types, dose, length, and adverse effects), therapeutic efficacy, relapses and therapeutic management, side effects, mean time of follow-up, therapies at the last visit, and death. The primary outcome measured was the rate of efficacy of first-line therapeutic approaches. Secondary outcomes measured included the rate of disease relapse, the outcome of untreated patients, the rate of patients without drug therapy at the end of follow-up, the rate of side effects, and mortality. Relapses were defined as a disease exacerbation following a period of improvement – whether or not the treatment response was complete.

For quality assessment, a prespecified study protocol was developed by BK and XB prior to the literature review, according to the MOOSE,^[8]^ AHRQ,^[9]^ STROBE,^[7]^ and GRACE^[10]^ recommendations/statements. Supplementary Table 1 summarizes the quality domains evaluated for each study. Possible overlapping data were managed by contrasting the following variables between studies: name of authors, participating centers, number of patients, epidemiological features, type of organ involvement, period of patient recruitment, and name of the database/multicenter group. When there was more than 1 report from the same group, we included only the publication having the most detailed therapeutic information for the entire cohort.

The ethical approval was not necessary because the study uses existing data (literature review).

### 2.4 Statistical analysis

Descriptive data were presented as means and standard deviation (SD) for continuous variables and numbers and percentages for categorical variables. Subset statistical analyses searching for potential sources of heterogeneity in the therapeutic approaches suggested by a previous study^[2]^ were carried out according to the geographical origin and the organ predominantly involved. The Chi-square test for contingency tables was used to compare gender, geographical origin, first-line regimens, therapeutic efficacy, relapses, side effects, mortality, and therapeutic management. Continuous outcomes such as mean age of the cohort, mean time of follow-up, and mean starting doses were compared using the nonparametric Kruskal–Wallis test. Forest plots with the odds ratios (ORs) and their 95% confidence intervals (CIs) were constructed to represent the association between study characteristics and outcomes. All significance tests were 2-tailed, and values of *P* < 0.05 were considered significant. All analyses were conducted by BK and NAD using the R V.3.2.3 for Windows statistical software package.

## 3 Results

**Figure 1:**
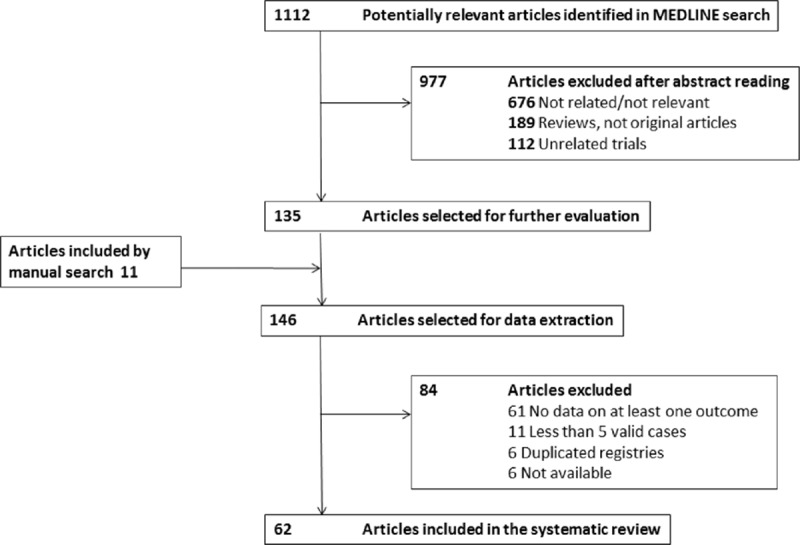
Results of the systematic search strategy.

The results of the systematic search strategy are summarized in Fig. 1: 62 studies^[11–72]^ including 3034 patients were analyzed (Supplementary Table 2). Table 1 summarizes the main patient characteristics.

**Table 1:**
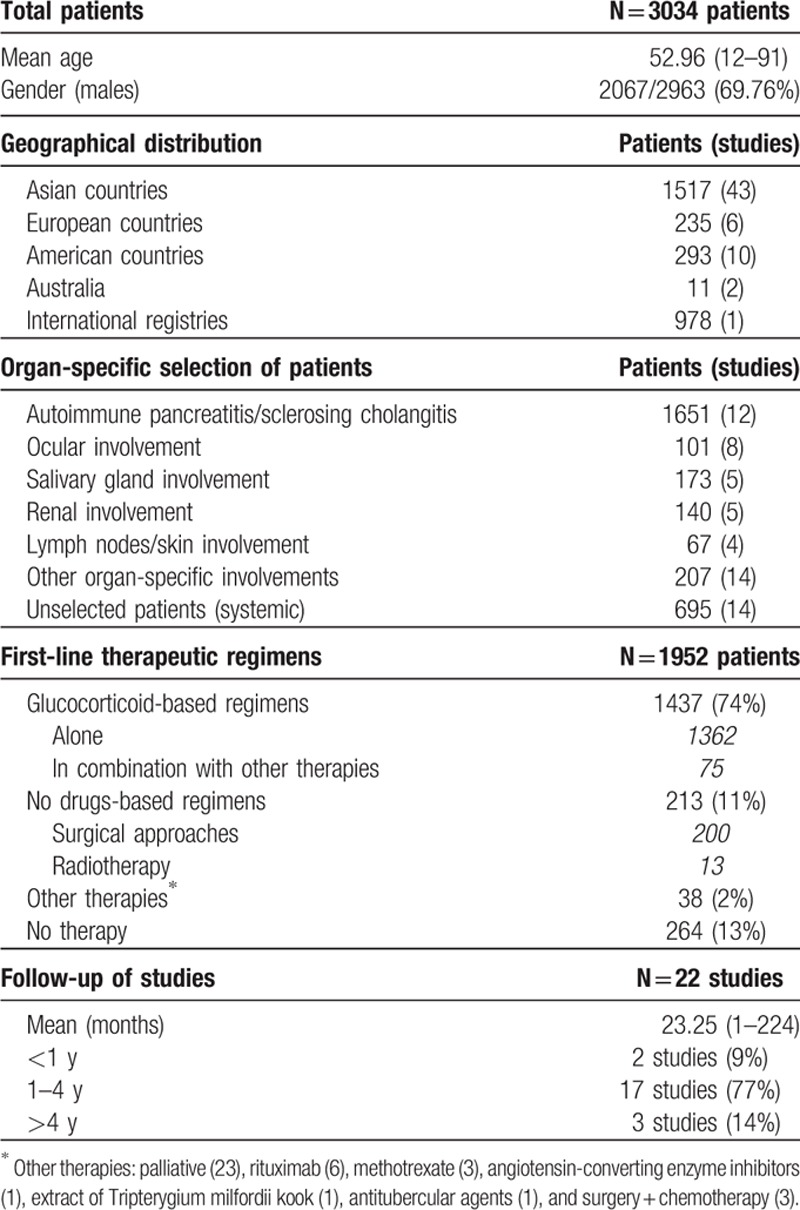
Results of the systematic search strategy: 62 studies including 3034 patients.

### 3.1 Outcomes

#### 3.1.1 Efficacy of first-line therapeutic approaches

Complete information about first-line therapeutic regimens was detailed in 1952 patients included in 48 studies (Table 1). First-line therapies included glucocorticoid-based regimens in 1437 (74%) patients, drug-free regimens (surgery or radiotherapy) in 213 (11%) patients, and other therapies in 38 (2%). No therapy, that is, wait-and-see management, was reported in 264 (13%) patients. Glucocorticoids were administered orally in all patients except for 10, who were treated with topical glucocorticoid preparations.

The mean starting dose was ≤0.6 mg/kg/d (equivalent to 40 mg/d ) in 24 (73%) of the 33 studies in which this information was provided. The efficacy of first-line therapies was detailed in 1293 patients, of whom 1246 (96%) were reported as having a therapeutic response. The efficacy of monotherapy with glucocorticoids was specified in 1220 patients, of whom 1186 (97%) were reported as having a therapeutic response. However, the glucocorticoids response was classified as complete in only 84/130 (65%) of the patients, partial/incomplete in 31/130 (24%), and absent (no response) in 15/130 (11%). The efficacy of other therapies was 14/17 (82%) for surgery, 20/22 (91%) for combined glucocorticoids/surgery, 17/22 (77%) for immunosuppressive/biological agents, and 9/12 (75%) for radiotherapy (Table 2).

**Table 2:**
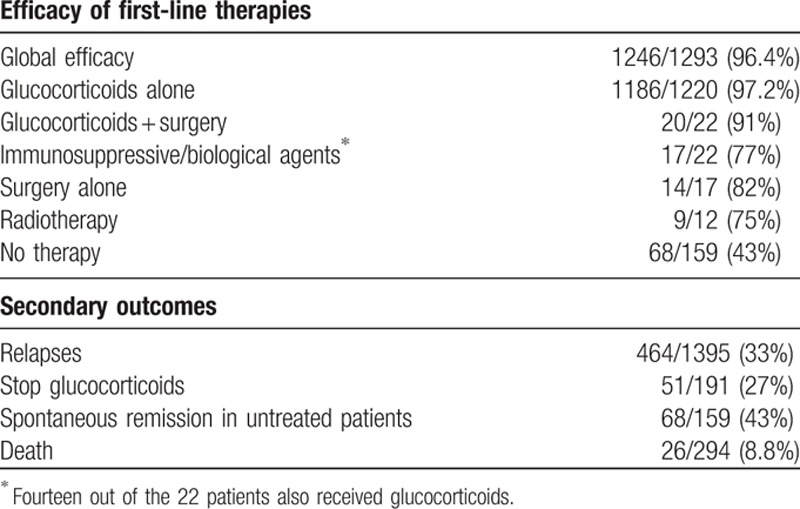
Efficacy of first-line therapeutic regimens and secondary outcomes (variables were not detailed in all studies, and the prevalence of a specific feature has been stated as number of cases with that feature/number of cases in which the feature was detailed).

Therapeutic failure with first-line therapies was reported in 47 patients (4%). The response to rescue therapies was detailed in only 18 cases and included glucocorticoids in 2 (response in both), surgery in 1 (no response), rituximab in 6 (response in 4), and immunosuppressants in 9 (response in 6) (Supplementary Table 3).

#### 3.1.2 Relapses

Relapses were reported in 464/1395 (33%) patients. The ongoing use of glucocorticoids at relapse was specified in 381 patients. Among these 381, the disease relapse occurred in 245 (64%) after the cessation of glucocorticoids. In more than one third of the patients, however (136; 36%), disease relapses occurred while patients were still receiving glucocorticoids.

Information on the therapeutic management of relapses was detailed in 378 cases and included mainly glucocorticoid use (either as a new course or as an increase/slow tapering of the ongoing dose) in 250 (66%) cases. The use of immunosuppressive agents was reported in 149 (39%) cases, principally in association with glucocorticoids (azathioprine was used in 126/149 cases). Rituximab use was reported in 9 (2%) cases. Therapeutic efficacy was reported in 219/231 (95%) of relapses treated with glucocorticoids, 56/69 (81%) of those treated with azathioprine, 16/22 (72%) of those treated with other immunosuppressive agents, and in all 9 cases treated with rituximab (100%).

#### 3.1.3 Side effects

Only 7 studies detailed the prevalence and/or types of side effects associated with therapies.^[12,18,24,49,50,66,70]^ Ebbo et al^[18]^ reported side effects related to glucocorticoids in 14/21 (67%) patients. Diabetes mellitus (DM) was the most frequently reported side effect in all studies. Other reported side effects included infections (n = 3), osteonecrosis (n = 2), psychosis (n = 1), vertebral fracture (n = 1), weight gain (n = 1), and hypertension (n = 1). Side effects related to azathioprine, reported in 17/49 (35%) patients, pertained primarily to gastrointestinal intolerance.^[70]^ There was also 1 case of azathioprine-induced pancreatitis.^[70]^

#### 3.1.4 Glucocorticoid cessation

Eleven studies detailed information on how many patients treated with glucocorticoids as first-line therapy were successful in discontinuing glucocorticoids completely at the time of the last visit. Only 51 (27%) of the 191 patients in whom this outcome was reported had been able to discontinue glucocorticoids entirely by the last visit. Three studies reported somewhat higher frequencies of glucocorticoid discontinuation (>50% of patients),^[19,54,73]^ but the relapse rates were higher in these studies compared to those in which patients remained on glucocorticoids. In summary, successful discontinuation of glucocorticoids for prolonged periods of time appears to be the exception rather than the rule for most patients with IgG4-RD.

#### 3.1.5 Outcome of untreated patients

In 14 studies, the authors detailed the outcome in 159 out of the 246 patients who did not receive treatment. Spontaneous improvement or resolution was reported in 68 (43%) cases, but long-term follow-up of the patients were seldom reported.

#### 3.1.6 Mortality

Information on survival was detailed in only 7 studies,^[16,18,19,24,50–52]^ which included a total of 294 patients. After a mean follow-up of 29.2 months (range 1–224 months), mortality was reported in 26 (8.8%) patients. The main causes of death included IgG4-RD progression (n = 7, including pulmonary disease in 4, aneurysm in 1, cholangitis in 1, and renal failure in 1) and cancer (n = 7). Other causes of death included cardiovascular disease (n = 4), infection (n = 3), and other/unknown (n = 5).

### 3.2 Association between study characteristics and outcomes

#### 3.2.1 Classification criteria bias

Four studies^[19,22,24,49]^ used classification criteria that include a positive response to glucocorticoids as one of the inclusion criteria (ICDC and HiSort criteria for autoimmune pancreatitis [AIP]); the efficacy of first-line glucocorticoid-based regimens was reported in 817/830 (98%) patients (no significant difference with respect to the remaining studies).

#### 3.2.2 Geographical bias

**Figure 2:**
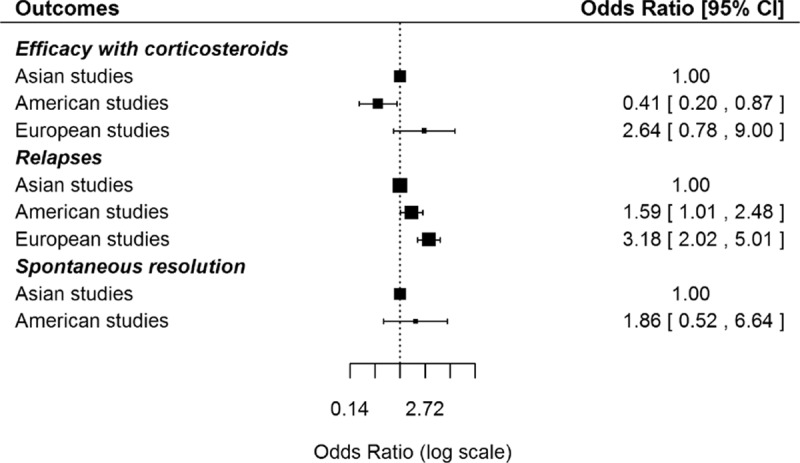
Differences in the main outcomes (efficacy of glucocorticoids, relapses, and spontaneous resolution) between Asian, American and European studies.

Table 3 summarizes the differences in the main baseline variables between Asian, American, and European studies. Epidemiologically, Asian studies included patients with a higher mean age (62 years vs 56.9 in American studies and 57.9 in European studies; *P* = 0.034). With respect to first-line therapeutic regimens, European studies more-frequently used glucocorticoids, American studies more-frequently used drug-free regimens, and Asian studies had the highest frequency of untreated patients (*P* < 0.001). With respect to outcomes (Fig. 2), American studies reported a lower efficacy (OR 0.41, 95% CI 0.20–0.87). Both American and European studies a higher frequency of relapses (OR 1.59, 95% CI 1.01–2.48 and OR 3.18, 95% CI 2.02–5.01, respectively) compared with Asian studies.

**Table 3:**
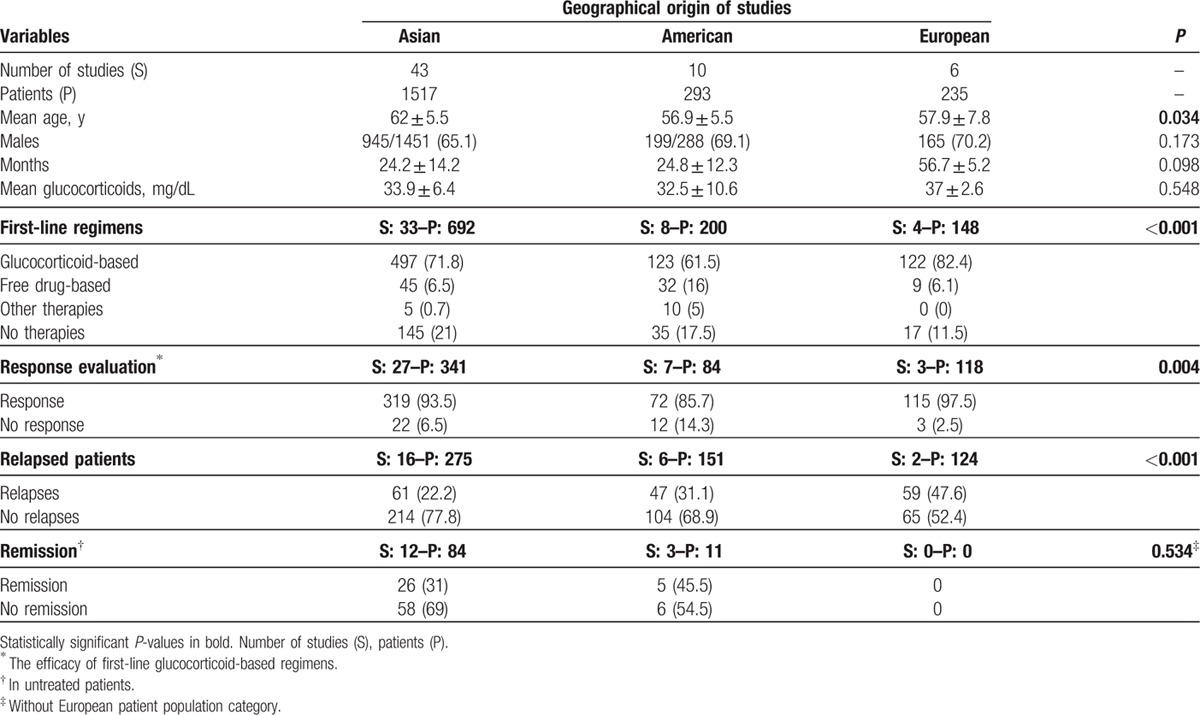
Differences in main baseline variables between Asian, American, and European studies.

#### 3.2.3 Organ-by-organ selection bias

**Figure 3:**
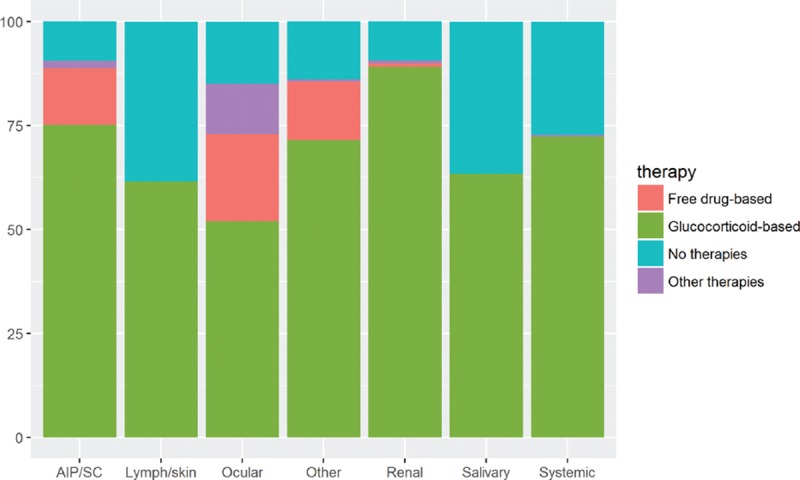
First-line therapeutic approaches used in the studies grouped according to the predominant organ involvement selected in each study (AIP = autoimmune pancreatitis, SC = sclerosing cholangitis).

**Figure 4:**
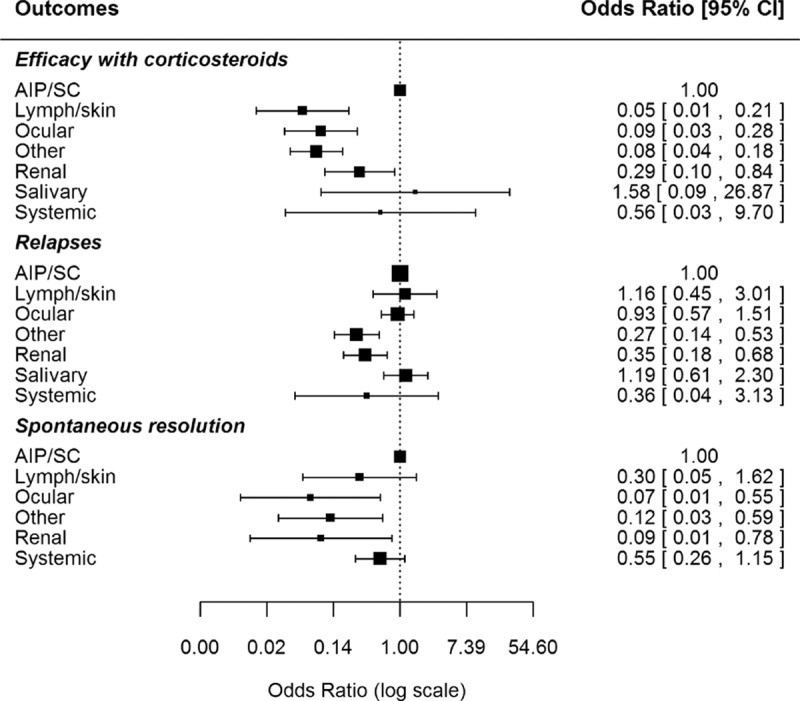
Differences in the main outcomes (efficacy of glucocorticoids, relapses, and spontaneous resolution) according to the predominant organ involvement selected in each study (AIP = autoimmune pancreatitis, SC = sclerosing cholangitis).

**Figure 5:**
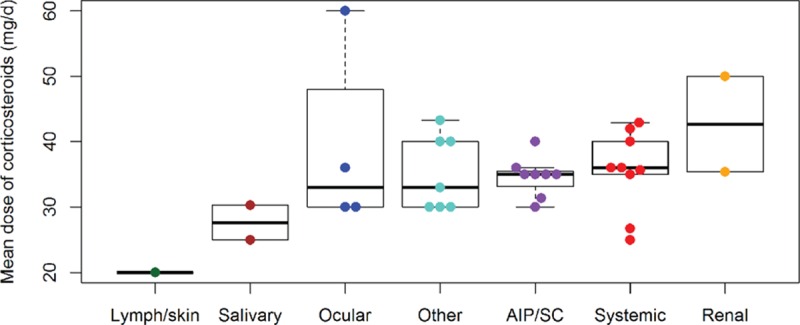
Differences in the mean dose of glucocorticoids used according to the predominant organ involvement selected in each study (AIP = autoimmune pancreatitis, SC = sclerosing cholangitis).

Table 4 compares the main variables according to the predominant organ involvement selected for each study. With regard to epidemiological features, we found significant differences with respect to the geographical (*P* < 0.001) and gender distribution (*P* < 0.001). In addition, wide heterogeneity was observed with respect to the first-line therapeutic approaches used for the different organ-specific subsets of patients (Fig. 3) and the outcomes reported (Fig. 4), including differences in the mean dose of glucocorticoids used for the different types of organ involvement (Fig. 5).

**Table 4:**
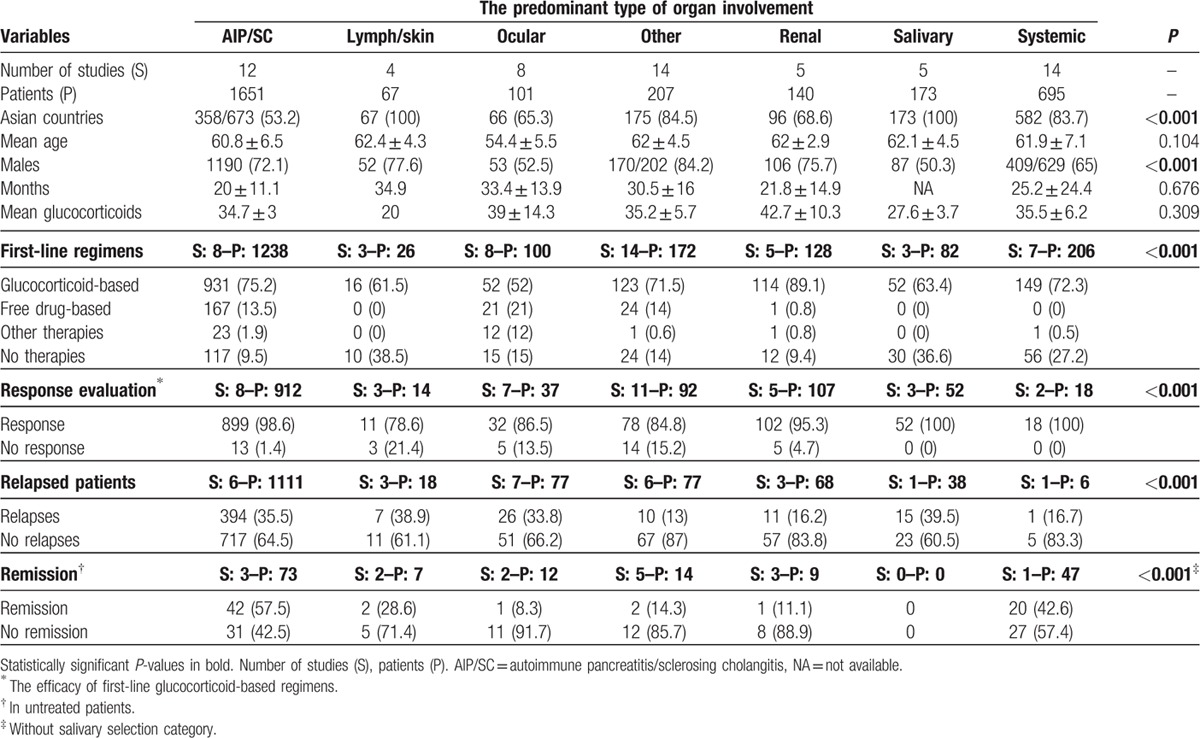
Comparison of the main variables according to the predominant involved organ selected for each study: AIP/SC, lymphadenopathy or skin involvement, ocular involvement, other involvements, renal involvement, salivary involvement, and systemic/multiorgan involvement.

## 4 Discussion

IgG4-RD, first described some 15 years ago, remains an emerging disease. The rapid increase in number of studies has mainly been due to descriptive characterizations of the disease. Few studies have been designed specifically to evaluate the therapeutic response, and information on treatment has, to date, been presented almost exclusively in descriptive studies rather than formal clinical trials. As a result, the evidence base on which to predicate therapeutic recommendations remains slim. Consequently, an evidence-based systematic review of published uncontrolled studies may provide the best available understanding of current treatment approaches. Although several reviews on IgG4-RD have recently been published,^[74,75]^ they did not specifically focus on the therapeutic management of the disease.

Since the first recognition of IgG4-RD as a discrete disease entity, glucocorticoid treatment has comprised the cornerstone of therapy for a majority of patients. Although such an approach is understandable given the affordability of this intervention and the substantial observational data that a majority of IgG4-RD patients respond to moderate to high doses of glucocorticoids, no controlled study has specifically evaluated their use. As a result, the optimal starting dose, the rate of glucocorticoid taper, the wisdom of discontinuing glucocorticoids altogether, patients’ long-term treatment response, and multiple other questions about this treatment approach remain uncertain.

The central place of glucocorticoids in the therapeutic armamentarium for IgG4-RD was extrapolated from observational studies in patients with type 1 AIP, in whom monotherapy with glucocorticoids has been largely uncontested as the first-line therapeutic approach. Our review confirms that glucocorticoid-based regimens were the therapeutic option of choice in the majority of patients (85%) in whom a specific therapeutic intervention was reported. A recent guideline recommends glucocorticoids as the first-line therapeutic agent, with 94% of interexpert agreement.^[4]^ This is supported by our findings: the efficacy of glucocorticoid-based first-line regimens was 97%. However, 4 major areas of uncertainty concerning the use of glucocorticoids in IgG4-RD persist: the definition of the induction dose, how to taper glucocorticoids, the duration of therapy, and the balance between efficacy and side effects.

The recent consensus guidelines^[4]^ recommend the use of prednisolone (0.6 mg/kg/d ) for 4 weeks as induction therapy. This mean dose was the most frequently used in our review (70% of studies), but varied widely according to geographical, epidemiological, and clinical features. Some studies^[41,68,69]^ used higher doses in patients with severe complications (pancreatic, pulmonary, renal, and retroperitoneal involvement), while others used lower doses in patients with diabetes, those at high risk of developing steroid-related side effects,^[24]^ or patients aged >80 years.^[69]^ Although the optimal starting glucocorticoids dose remains undefined, it appears that doses equivalent to <10 mg/d of prednisone are associated with a lack of response.^[55]^

After the first 4 weeks of induction therapy, the glucocorticoid dose can be tapered gradually.^[4]^ One approach, frequently employed in Japan, is to taper glucocorticoids over several months to a daily dose equivalent of somewhere between 2.5 and 10.0 mg/d of prednisone and then to maintain this dose for several years. This approach has been developed through consensus^[76]^ rather than through rigorous, controlled studies, however, and its long-term efficacy has not been evaluated prospectively. A retrospective, multicenter study of 459 AIP patients in Japan reported that 82% of patients still received glucocorticoids as maintenance therapy at the last visit.^[77]^ An alternative approach to the use of glucocorticoids, commonly employed in North America, is to taper prednisone to discontinuation after 2 to 3 months and to add a steroid-sparing agent if the disease relapses during or after the taper. The strategy acknowledges the facts that glucocorticoids do not cure IgG4-RD and the long-term morbidity of glucocorticoid use may be substantial in this patient population, which often has a number of comorbidities concomitantly with IgG4-RD.

Although the goal should probably be discontinuation of glucocorticoids after achieving a maintained clinical response, we found this only happened in 25% of patients in whom information on therapy at the last visit was detailed.

A key finding of this review is the very limited reported information on the side effects associated with widespread glucocorticoid use. Only one small series detailed the frequency of glucocorticoid-related side effects, which were found in two thirds of patients. DM is the most frequently reported side effect.^[51]^ A detailed study by Ito et al evaluated the need for DM therapies after glucocorticoid treatment in 22 IgG4-RD patients without AIP and found that the percentage of untreated patients was reduced from 72% to 50% after starting glucocorticoids, while the rate of patients requiring insulin therapy increased from 4% to 27%; in addition, 83% of patients were aged >50 years and diagnosed with DM or impaired glucose tolerance.

The selection of a “steroid-sparing” agent is challenged by the paucity of data on the efficacy of conventional agents for IgG4-RD. Conventional immunomodulatory agents have been reported as first-line therapies in fewer than 10 patients. The recent therapeutic consensus^[4]^ reported differing clinical practice according to country, with more Japanese physicians supporting glucocorticoid monotherapy as the first-line option compared with experts from other countries (North America, Europe, Korea, and China). However, in our geographical analysis, Asian studies had the highest frequency of untreated patients while glucocorticoids were more frequently used in Europeans and drug-free treatment in US patients: surprizingly, the rate of relapses was lower in the Asian population. This might be explained by taking into account the fact that Asian studies did not detail information on relapses (missing information would provide biased results) and because Asian studies included a lower frequency of patients affected by AIP (AIP is one of the IgG4-related involvements with the highest rate of relapses).

There is widespread consensus on the use of immunosuppressive agents as steroid-sparing agents in refractory/complicated cases. We found that immunosuppressants were used in nearly 40% of cases who relapsed but that these agents were employed overwhelmingly in combination with glucocorticoids, complicating any assessment of their efficacy alone. Azathioprine was used in 85% of cases, probably reflecting clinical practice in cases of AIP, followed by mycophenolate mofetil, methotrexate, tacrolimus, and cyclophosphamide. No study has compared the efficacy of different immunosuppressive agents. The efficacy of the azathioprine/glucocorticoid combination was estimated to be on the order of 80%. Some patients relapsed when treated with low doses of azathioprine (50 mg/d) or mycophenolate (1 g/d) and the disease was controlled by increasing the dose.^[19]^ This was probably in fact azathioprine coupled simultaneously with an increase in prednisone dose, making it difficult to be certain about how much improvement was due to azathioprine and how much to increased steroids. Interpretation of the efficacy of all conventional agents in IgG4-RD is hampered by the dearth of prospective, controlled experiences with these medications. In addition, most data describing the use of conventional immunosuppressive medications are confounded by concomitant glucocorticoid use.

The emergence of biological therapies has increased the therapeutic armamentarium available for treating the most refractory/severe cases of IgG4-RD, but their use is limited by the lack of licensing. Rituximab was the most-reported biological option in our systematic review, being used in 9 studies: it was used less frequently as induction therapy (only 8 cases with an efficacy of 75%)^[20,64]^ and more frequently as rescue therapy in patients who failed to achieve or sustain disease remission with glucocorticoid treatment.^[11,18,22,31,32,49,66]^ Rituximab use was first reported in 2010^[78]^ and was then used especially in patients with orbital involvement.^[64,66]^ Carruthers et al^[79]^ have recently published the results of an open-label prospective study including 30 IgG4-RD patients treated with 2 doses of rituximab (1000 mg each), with a disease response rate of 97%. The primary outcome was achieved by 23 participants (77%): 14 (47%) were in complete remission at 6 months, and 12 (40%) remained in complete remission at 12 months. These effects were achieved largely without glucocorticoid use (26 of the 30 patients were not treated with glucocorticoids) and without readministration of rituximab for the purpose of remission maintenance. Yamamoto et al^[80]^ have also reported the successful use of rituximab without associated glucocorticoids. Further studies are needed to better define the place of rituximab in the therapeutic approach to IgG4-RD: although currently considered a rescue therapy, it might be considered as a first-line option in some cases. The apparent success of B cell depletion strategies has raised interest in other treatment strategies targeting the B cell lineage. A trial of Xmab5871, a homodimer that binds simultaneously to CD19 and Fc-gammaRIIIb – leading in theory to inhibition of the targeted cell – is now under study.

Fifteen percent of first-line therapeutic interventions in IgG4-RD were glucocorticoid-free regimens: these were mainly surgical (11% of the total) with radiotherapy used in only 13 cases. As a cardinal feature of IgG4-RD is single or multiple organ swelling that often raises concerns about malignancy, surgery is mainly reported to treat masses located in the pancreas, kidneys, lungs, biliary tract, and prostate. In these patients, IgG4-RD is diagnosed accidentally after surgical removal of a potential tumor. Most patients were retrospectively diagnosed with IgG4-RD based on histopathologic findings. The best examples are: presumed cholangiocarcinoma,^[19]^ pancreatic cancer,^[19]^ pulmonary or pleural tumors,^[72]^ prostate adenocarcinoma,^[13]^ or renal cancer.^[50]^

On the other hand, an IgG4-specific surgical approach has also been used in patients with specific involvements (mainly infiltrative masses that involve tubular anatomical structures and may lead to obstructive processes). The best examples are: dilatation/stenting of the biliary tract in patients with IgG4-related sclerosing cholangitis,^[49,62]^ ureteral stents/ureterolysis,^[32]^ transurethral resection of the prostate/suprapubic prostatectomy,^[13]^ ocular surgical excision,^[53,64]^ or surgical resection of submaxillary tumors.^[34]^ In these cases, long-standing highly fibrotic lesions may respond poorly to drug-based therapies, and surgical debulking may be an appropriate and useful option.

A further key finding of our review was the lack of any therapeutic intervention in 13% of patients. In some specific organ involvements, this percentage was higher, reaching 71% in IgG4-related lymphadenopathy, 35% in IgG4-related salivary involvement, or 40% in IgG4-related skin involvement. Eight studies^[18,25,29,41,46,54,67,72]^ detailed the reasons in 54 cases, which included mild/residual involvement or the patient refusing to be treated (n = 50), severe DM (n = 3), and active tuberculosis infection (n = 1). The outcome of these patients shows relapse or progression in nearly 60% of cases, a rate 2-fold higher than that found for patients treated with GC-therapeutic regimens. Wait and see management may be appropriate in asymptomatic patients with lymphadenopathy, cutaneous features, or mild salivary gland enlargement.

Another key finding of our review is the widespread lack of a standardized method of evaluating the therapeutic response. Of the 48 studies that evaluated the efficacy of first-line therapy, a specific definition of the type of therapeutic response was detailed in only 25. Only 2 studies^[18,19]^ used an objective score; the remaining 23 used a subjective evaluation including clinical, laboratory, imaging, and/or organ-specific diagnostic tests (10 studies used ≥2 items and 13 only 1 item). Only 4 studies detailed reductions in serum IgG4 levels as a parameter of therapeutic efficacy. The development and validation of rigorous outcome measures are an important goal for the field of IgG4-RD investigation. An international effort to validate the IgG4-RD Responder Index^[81]^ is now under way.

Recent studies have investigated novel cellular and molecular pathways involved in the pathogenesis of IgG4-RD that may be therapeutically targeted by biological therapies, including a potential role for basophil activation mediated by IgE or by Toll-like receptors.^[82]^ Clayton et al^[83]^ tested the use of omalizumab (biological therapy against IgE) in patients with IgG4-related eosinophilic esophagitis, while other studies^[84,85]^ have found a raised expression of some cytokines, such as IL10 and Blys/BAFF, opening a possible therapeutic approach by targeting these molecules.

IgG4-RD is an increasingly recognized condition in adults, with a heterogeneous clinical presentation affecting a wide range of organ systems. The principal bases of therapeutic decision-making currently remain clinical experience and expert opinion, due to the low level of evidence with respect to therapeutic data. The body of evidence relies predominantly on descriptive series including a range of 5 to 50 patients (74% of studies). Our review found that although nearly 70% of reported IgG4-RD patients are treated with monotherapy with oral glucocorticoids, there is wide heterogeneity and therapeutic management is heavily influenced by geographical, epidemiological, and clinical factors, especially with respect to the predominant organ affected by the disease. International efforts are required to collect and characterize large multicenter and multidisciplinary cohorts of patients in order to develop consensual therapeutic approaches and endpoints.

## Supplementary Material

Supplemental Digital Content

## References

[1] Stone JH Zen Y Deshpande V IgG4-related disease. *N Engl J Med* 2012; 366:539–551. 22316447 10.1056/NEJMra1104650

[2] Brito-Zeron P Ramos-Casals M Bosch X The clinical spectrum of IgG4-related disease. *Autoimmun Rev* 2014; 13:1203–1210. 25151972 10.1016/j.autrev.2014.08.013

[3] Stone JH Brito-Zeron P Bosch X Diagnostic approach to the complexity of IgG4-related disease. *Mayo Clin Proc* 2015; 90:927–939. 26141331 10.1016/j.mayocp.2015.03.020

[4] Khosroshahi A Wallace ZS Crowe JL International consensus guidance statement on the management and treatment of IgG4-related disease. *Arthritis Rheumatol (Hoboken, NJ)* 2015; 67:1688–1699. 10.1002/art.39132 25809420

[5] Shea BJ Grimshaw JM Wells GA Development of AMSTAR: a measurement tool to assess the methodological quality of systematic reviews. *BMC Med Res Methodol* 2007; 7:10. 17302989 10.1186/1471-2288-7-10 PMC1810543

[6] Masaki Y Shimizu H Sato Nakamura T IgG4-related disease: diagnostic methods and therapeutic strategies in Japan. *J Clin Exp Hematop* 2014; 54:95–101. 25318941 10.3960/jslrt.54.95

[7] von Elm E Altman DG Egger M The Strengthening the Reporting of Observational Studies in Epidemiology (STROBE) Statement: guidelines for reporting observational studies. *Lancet* 2007; 370:1453–1457. 18064739 10.1016/S0140-6736(07)61602-X

[8] Manchikanti L Datta S Smith HS Evidence-based medicine, systematic reviews, and guidelines in interventional pain management: part 6. Systematic reviews and meta-analyses of observational studies. *Pain Physician* 2009; 12:819–850. 19787009

[9] Shamliyan T Kane RL Jansen S Quality of systematic reviews of observational nontherapeutic studies. *Prev Chronic Dis* 2010; 7:A133. 20950540 PMC2995597

[10] Dreyer NA Schneeweiss S McNeil BJ GRACE principles: recognizing high-quality observational studies of comparative effectiveness. *Am J Manag Care* 2010; 16:467–471. 20560690

[11] Alexander MP Larsen CP Gibson IW Membranous glomerulonephritis is a manifestation of IgG4-related disease. *Kidney Int* 2013; 83:455–462. 23254897 10.1038/ki.2012.382

[12] Bosco JJ Suan D Varikatt W Lin MW Extra-pancreatic manifestations of IgG4-related systemic disease: a single-centre experience of treatment with combined immunosuppression. *Intern Med J* 2013; 43:417–423. 23013529 10.1111/j.1445-5994.2012.02964.x

[13] Buijs J Maillette de Buy Wenniger L van Leenders G Immunoglobulin G4-related prostatitis: a case-control study focusing on clinical and pathologic characteristics. *Urology* 2014; 83:521–526. 24581512 10.1016/j.urology.2013.10.052

[14] Carruthers MN Khosroshahi A Augustin T The diagnostic utility of serum IgG4 concentrations in IgG4-related disease. *Ann Rheum Dis* 2014:14–18. 24651618 10.1136/annrheumdis-2013-204907

[15] Chen H Lin W Wang Q IgG4-related disease in a Chinese cohort: a prospective study. *Scand J Rheumatol* 2014; 43:70–74. 24134471 10.3109/03009742.2013.822094

[16] Cheuk W Yuen HKL Chu SYY Lymphadenopathy of IgG4-related sclerosing disease. *Am J Surg Pathol* 2008; 32:671–681. 18344866 10.1097/PAS.0b013e318157c068

[17] Detlefsen S Zamboni G Frulloni L Clinical features and relapse rates after surgery in type 1 autoimmune pancreatitis differ from type 2: a study of 114 surgically treated European patients. *Pancreatology* 2012; 12:276–283. 22687385 10.1016/j.pan.2012.03.055

[18] Ebbo M Daniel L Pavic M IgG4-related systemic disease: features and treatment response in a French cohort: results of a multicenter registry. *Medicine (Baltimore)* 2012; 91:49–56. 22198501 10.1097/MD.0b013e3182433d77

[19] Ghazale A Chari ST Zhang L Immunoglobulin G4-associated cholangitis: clinical profile and response to therapy. *Gastroenterology* 2008; 134:706–715. 18222442 10.1053/j.gastro.2007.12.009

[20] Ginat DT Freitag SK Kieff D Radiographic patterns of orbital involvement in IgG4-related disease. *Ophthal Plast Reconstr Surg* 2013; 29:261–266. 10.1097/IOP.0b013e31829165ad 23645355

[21] Go H Kim JE Kim Ya Ocular adnexal IgG4-related disease: comparative analysis with mucosa-associated lymphoid tissue lymphoma and other chronic inflammatory conditions. *Histopathology* 2012; 60:296–312. 22211288 10.1111/j.1365-2559.2011.04089.x

[22] Hart PA Kamisawa T Brugge WR Long-term outcomes of autoimmune pancreatitis: a multicentre, international analysis. *Gut* 2013; 62:1771–1776. 23232048 10.1136/gutjnl-2012-303617 PMC3862979

[23] Hirano K Tada M Sasahira N Incidence of malignancies in patients with IgG4-related disease. *Intern Med* 2014; 53:171–176. 24492683 10.2169/internalmedicine.53.1342

[24] Huggett MT Culver EL Kumar M Type 1 autoimmune pancreatitis and IgG4-related sclerosing cholangitis is associated with extrapancreatic organ failure, malignancy, and mortality in a prospective UK cohort. *Am J Gastroenterol* 2014; 109:1675–1683. 25155229 10.1038/ajg.2014.223 PMC4552254

[25] Inoue D Zen Y Abo H Immunoglobulin G4-related periaortitis and periarteritis: CT findings in 17 patients. *Radiology* 2011; 261:625–633. 21803920 10.1148/radiol.11102250

[26] Inoue D Zen Y Sato Y IgG4-related perineural disease. *Int J Rheumatol* 2012; 2012:401890. 22523496 10.1155/2012/401890 PMC3317227

[27] Ito N Yagi K Kawano M Analysis of pancreatic endocrine function in patients with IgG4-related diseases, in whom autoimmune pancreatitis was ruled out by diagnostic imaging. *Endocr J* 2014; 61:765–772. 24882517 10.1507/endocrj.ej14-0078

[28] Kamisawa T Takuma K Tabata T Serum IgG4-negative autoimmune pancreatitis. *J Gastroenterol* 2011; 46:108–116. 20824290 10.1007/s00535-010-0317-2

[29] Katsura M Mori H Kunimatsu A Radiological features of IgG4-related disease in the head, neck, and brain. *Neuroradiology* 2012; 54:873–882. 22358111 10.1007/s00234-012-1012-1

[30] Kawano M Saeki T Nakashima H Proposal for diagnostic criteria for IgG4-related kidney disease. *Clin Exp Nephrol* 2011; 15:615–626. 21898030 10.1007/s10157-011-0521-2

[31] Khosroshahi A Carruthers MN Deshpande V Rituximab for the treatment of IgG4-related disease: lessons from 10 consecutive patients. *Medicine (Baltimore)* 2012; 91:57–66. 22210556 10.1097/MD.0b013e3182431ef6

[32] Khosroshahi A Carruthers MN Stone JH Rethinking Ormond's disease: “idiopathic” retroperitoneal fibrosis in the era of IgG4-related disease. *Medicine (Baltimore)* 2013; 92:82–91. 23429355 10.1097/MD.0b013e318289610f PMC4553983

[33] Kim TY Park KS Choi JS Comparative clinical manifestations of IgG4- related and IgG4-negative primary tubulointerstitial nephritis. *Clin Nephrol* 2011; 76:440–446. 22105446 10.5414/cn107117

[34] Kitagawa S Zen Y Harada K Abundant IgG4-positive plasma cell infiltration characterizes chronic sclerosing sialadenitis (Küttner's tumor). *Am J Surg Pathol* 2005; 29:783–791. 15897744 10.1097/01.pas.0000164031.59940.fc

[35] Kiyama K Kawabata D Hosono Y Serum BAFF and APRIL levels in patients with IgG4-related disease and their clinical significance. *Arthritis Res Ther* 2012; 14:R86. 22531553 10.1186/ar3810 PMC3446460

[36] Koizumi S Kamisawa T Kuruma S Clinical features of IgG4-related dacryoadenitis. *Graefes Arch Clin Exp Ophthalmol* 2014; 252:491–497. 24318531 10.1007/s00417-013-2541-y

[37] Kubota T Moritani S Ocular adnexal IgG4-related lymphoplasmacytic infiltrative disorder. *Arch Ophthalmol* 2010; 128:577–584. 20457978 10.1001/archophthalmol.2010.45

[38] Kuruma S Kamisawa T Tabata T Clinical characteristics of patients with autoimmune pancreatitis with or without Mikulicz's disease and Mikulicz's disease alone. *Gut Liver* 2013; 7:96–99. 23422705 10.5009/gnl.2013.7.1.96 PMC3572327

[39] Laco J Podhola M Kamarádová K Idiopathic vs. secondary retroperitoneal fibrosis: a clinicopathological study of 12 cases, with emphasis to possible relationship to IgG4-related disease. *Virchows Arch* 2013; 463:721–730. 24052251 10.1007/s00428-013-1480-7

[40] Lindstrom KM Cousar JB Lopes MBS IgG4-related meningeal disease: clinico-pathological features and proposal for diagnostic criteria. *Acta Neuropathol* 2010; 120:765–776. 20844883 10.1007/s00401-010-0746-2

[41] Masaki Y Dong L Kurose N Proposal for a new clinical entity, IgG4-positive multiorgan lymphoproliferative syndrome: analysis of 64 cases of IgG4-related disorders. *Ann Rheum Dis* 2009; 68:1310–1315. 18701557 10.1136/ard.2008.089169

[42] Matsubayashi H Sawai H Kimura H Characteristics of autoimmune pancreatitis based on serum IgG4 level. *Dig Liver Dis* 2011; 43:731–735. 21515099 10.1016/j.dld.2011.03.006

[43] Matsui S Hebisawa A Sakai F Immunoglobulin G4-related lung disease: clinicoradiological and pathological features. *Respirology* 2013; 18:480–487. 23145930 10.1111/resp.12016

[44] Matsuo T Ichimura K Sato Y Immunoglobulin G4 (IgG4)-positive or -negative ocular adnexal benign lymphoid lesions in relation to systemic involvement. *J Clin Exp Hematop* 2010; 50:129–142. 21123971 10.3960/jslrt.50.129

[45] Mizushima I Inoue D Yamamoto M Clinical course after corticosteroid therapy in IgG4-related aortitis/periaortitis and periarteritis: a retrospective multicenter study. *Arthritis Res Ther* 2014; 16:R156. 25056443 10.1186/ar4671 PMC4220557

[46] Moteki H Yasuo M Hamano H IgG4-related chronic rhinosinusitis: a new clinical entity of nasal disease. *Acta Otolaryngol* 2011; 131:518–526. 21162659 10.3109/00016489.2010.533699 PMC3490482

[47] Oh H-C Kim M-H Lee KT Clinical clues to suspicion of IgG4-associated sclerosing cholangitis disguised as primary sclerosing cholangitis or hilar cholangiocarcinoma. *J Gastroenterol Hepatol* 2010; 25:1831–1837. 21091993 10.1111/j.1440-1746.2010.06411.x

[48] Ohta N Kurakami K Ishida A Clinical and pathological characteristics of IgG4-related sclerosing sialadenitis. *Laryngoscope* 2012; 122:572–577. 22241660 10.1002/lary.22449

[49] Patel H Khalili K Kyoung KT IgG4 related disease – a retrospective descriptive study highlighting Canadian experiences in diagnosis and management. *BMC Gastroenterol* 2013; 13:168. 24321047 10.1186/1471-230X-13-168 PMC3878912

[50] Raissian Y Nasr SH Larsen CP Diagnosis of IgG4-related tubulointerstitial nephritis. *J Am Soc Nephrol* 2011; 22:1343–1352. 21719792 10.1681/ASN.2011010062 PMC3137582

[51] Saeki T Kawano M Mizushima I The clinical course of patients with IgG4-related kidney disease. *Kidney Int* 2013; 84:826–833. 23698232 10.1038/ki.2013.191

[52] Sakamoto A Nagai R Saito K Idiopathic retroperitoneal fibrosis, inflammatory aortic aneurysm, and inflammatory pericarditis-retrospective analysis of 11 case histories. *J Cardiol* 2012; 59:139–146. 22154614 10.1016/j.jjcc.2011.07.014

[53] Sato Y Ohshima KI Ichimura K Ocular adnexal IgG4-related disease has uniform clinicopathology. *Pathol Int* 2008; 58:465–470. 18705764 10.1111/j.1440-1827.2008.02257.x

[54] Sato Y Inoue D Asano N Association between IgG4-related disease and progressively transformed germinal centers of lymph nodes. *Mod Pathol* 2012; 25:956–967. 22481280 10.1038/modpathol.2012.54

[55] Sato Y Takeuchi M Takata K Clinicopathologic analysis of IgG4-related skin disease. *Mod Pathol* 2013; 26:523–532. 23174935 10.1038/modpathol.2012.196

[56] Suzuki M Nakamaru Y Akazawa S Nasal manifestations of immunoglobulin G4-related disease. *Laryngoscope* 2013; 123:829–834. 23168498 10.1002/lary.23792

[57] Tabata T Kamisawa T Takuma K Serial changes of elevated serum IgG4 levels in IgG4-related systemic disease. *Intern Med* 2011; 50:69–75. 21245628 10.2169/internalmedicine.50.4321

[58] Takahashi H Yamashita H Morooka M The utility of FDG-PET/CT and other imaging techniques in the evaluation of IgG4-related disease. *Jt Bone Spine* 2014; 81:331–336. 10.1016/j.jbspin.2014.01.010 24568886

[59] Takagi Y Nakamura H IgG4-related Mikulicz’ s disease: ultrasonography of the salivary and lacrimal glands for monitoring the efficacy of corticosteroid therapy. *Clin Exp Rheumatol* 2013; 773–775. 23806290

[60] Takagi D Nakamaru Y Fukuda S Otologic manifestations of immunoglobulin G4-related disease. *Ann Otol Rhinol Laryngol* 2014; 123:420–424. 24682733 10.1177/0003489414526844

[61] Takuma K Kamisawa T Anjiki H Metachronous extrapancreatic lesions in autoimmune pancreatitis. *Intern Med* 2010; 49:529–533. 20228586 10.2169/internalmedicine.49.3038

[62] Tanaka A Tazuma S Okazaki K Nationwide survey for primary sclerosing cholangitis and IgG4-related sclerosing cholangitis in Japan. *J Hepatobiliary Pancreat Sci* 2014; 21:43–50. 24353071 10.1002/jhbp.50

[63] Triantopoulou C Malachias G Maniatis P Renal lesions associated with autoimmune pancreatitis: CT findings. *Acta Radiol* 2010; 51:702–707. 20429758 10.3109/02841851003738846

[64] Wallace ZS Deshpande V Stone JH Ophthalmic manifestations of IgG4-related disease: single-center experience and literature review. *Semin Arthritis Rheum* 2014; 43:806–817. 24513111 10.1016/j.semarthrit.2013.11.008

[65] Watanabe T Maruyama M Ito T Clinical features of a new disease concept, IgG4-related thyroiditis. *Scand J Rheumatol* 2013; 42:325–330. 23496326 10.3109/03009742.2012.761281

[66] Wu A Andrew NH Tsirbas A Rituximab for the treatment of IgG4-related orbital disease: experience from five cases. *Eye (Lond)* 2015; 29:122–128. 25341435 10.1038/eye.2014.251 PMC4289839

[67] Yamada K Hamaguchi Y Saeki T Investigations of IgG4-related disease involving the skin. *Mod Rheumatol* 2013; 23:986–993. 23111461 10.1007/s10165-012-0786-7

[68] Yamamoto M Takahashi H Ishigami K Relapse patterns in IgG4-related disease. *Ann Rheum Dis* 2012; 71:1755–11755. 22589378 10.1136/annrheumdis-2012-201694

[69] Yamamoto M Nojima M Takahashi H Identification of relapse predictors in IgG4-related disease using multivariate analysis of clinical data at the first visit and initial treatment. *Rheumatology (Oxford)* 2015; 54:45–49. 24907151 10.1093/rheumatology/keu228

[70] Yoo JJ Park JJ Kang EH Risk factors for the recurrence of IgG 4-related sclerosing disease without autoimmune pancreatitis. *J Clin Rheumatol* 2011; 17:392–394. 21946471 10.1097/RHU.0b013e31823262d5

[71] You M-W Kim JH Byun JH Relapse of IgG4-related sclerosing cholangitis after steroid therapy: image findings and risk factors. *Eur Radiol* 2014; 24:1039–1048. 24573568 10.1007/s00330-014-3127-8

[72] Zen Y Inoue D Kitao A IgG4-related lung and pleural disease: a clinicopathologic study of 21 cases. *Am J Surg Pathol* 2009; 33:1886–1893. 19898222 10.1097/PAS.0b013e3181bd535b

[73] Khosroshahi A Carruthers MN Deshpande V Rituximab for the treatment of IgG4-related disease: lessons from 10 consecutive patients. *Medicine (Baltimore)* 2012; 91:57–66. 22210556 10.1097/MD.0b013e3182431ef6

[74] Karim AF Verdijk RM Guenoun J An inflammatory condition with different faces: immunoglobulin G4-related disease. *Neth J Med* 2016; 74:110–115. 27020990

[75] Mulholland GB Jeffery CC Satija P Immunoglobulin G4-related diseases in the head and neck: a systematic review. *J Otolaryngol Head Neck Surg* 2015; 44:24. 26092582 10.1186/s40463-015-0071-9 PMC4482182

[76] Kamisawa T Okazaki K Kawa S Japanese consensus guidelines for management of autoimmune pancreatitis: III. Treatment and prognosis of AIP. *J Gastroenterol* 2010; 45:471–477. 20213336 10.1007/s00535-010-0221-9

[77] Kamisawa T Shimosegawa T Okazaki K Standard steroid treatment for autoimmune pancreatitis. *Gut* 2009; 58:1504–1507. 19398440 10.1136/gut.2008.172908

[78] Khosroshahi A Bloch DB Deshpande V Rituximab therapy leads to rapid decline of serum IgG4 levels and prompt clinical improvement in IgG4-related systemic disease. *Arthritis Rheum* 2010; 62:1755–1762. 20191576 10.1002/art.27435

[79] Carruthers MN Topazian MD Khosroshahi A Rituximab for IgG4-related disease: a prospective, open-label trial. *Ann Rheum Dis* 2015; 74:1171–1177. 25667206 10.1136/annrheumdis-2014-206605

[80] Yamamoto M Awakawa T Takahashi H Is rituximab effective for IgG4-related disease in the long term? Experience of cases treated with rituximab for 4 years. *Ann Rheum Dis* 2015; 74:e46. 25862615 10.1136/annrheumdis-2015-207625

[81] Carruthers MN Stone JH Deshpande V Development of an IgG4-RD responder index. *Int J Rheumatol* 2012; 2012:259408. 22611406 10.1155/2012/259408 PMC3348627

[82] Sharma M Bayry J Autoimmunity: basophils in autoimmune and inflammatory diseases. *Nat Rev Rheumatol* 2015; 11:129–131. 25422004 10.1038/nrrheum.2014.199

[83] Clayton F Fang JC Gleich GJ Eosinophilic esophagitis in adults is associated with IgG4 and not mediated by IgE. *Gastroenterology* 2014; 147:602–609. 24907494 10.1053/j.gastro.2014.05.036

[84] Furukawa S Moriyama M Tanaka A Preferential M2 macrophages contribute to fibrosis in IgG4-related dacryoadenitis and sialoadenitis, so-called Mikulicz's disease. *Clin Immunol* 2015; 156:9–18. 25450336 10.1016/j.clim.2014.10.008

[85] Lin W Jin L Chen H B cell subsets and dysfunction of regulatory B cells in IgG4-related diseases and primary Sjogren's syndrome: the similarities and differences. *Arthritis Res Ther* 2014; 16:R118. 24887143 10.1186/ar4571 PMC4075418

