# Supplementary material for: Therapeutic approach to IgG4-related disease: A systematic review
Source: Medicine (Baltimore). 2016 Jul 1;95(26):e4002. doi: 10.1097/MD.0000000000004002 (PMC4937924; doi:10.1097/MD.0000000000004002)
Supplement: Supplemental Digital Content [file medi-95-e4002-s001.doc]

**Supplementary TABLE 1.** Protocol for quality assessment of observational therapeutic studies in IgG4-RD: quality domains analyzed

1. **Sample definition and selection**
   1. Epidemiological profile (mean age at dx, range, gender, country of study)
   2. Inclusion criteria (diagnostic criteria)
   3. Exclusion criteria
   4. Clinical subsets (systemic vs organ-specific)
   5. Sample size
2. **Intervention**
   1. Study design (interventional/descriptive)
   2. Drug-based therapies (drugs, dose, length)
   3. Non-drug therapeutic approaches
   4. First/second line strategies
   5. Isolated or combined interventions
3. **Outcomes**
   1. Primary (efficacy of therapy) and secondary (relapses, mortality)
   2. Definition
4. **Soundness of information**
   1. Intervention: center/centers, period of recruitment, ethical issues, informed consent
   2. Outcome: direct or indirect evaluation
5. **Follow-up**
   1. Mean, range
   2. Missing data
6. **Analysis**
   1. Indication of therapy (severity)
   2. Intention-to-treat, items specified a priori
   3. Maintenance therapy
   4. Relapses
   5. Side effects
   6. Survival
   7. Analysis of covariates (stratification, multivariate, propensity, sensitivity analysis)
   8. Intra-group comparisons

**Supplementary TABLE 2.** List of manuscripts included in the systematic review: main features.

| Reference | First author | Year | Patients | Country | Predominant organ involvement | Patients with therapeutic data detailed |
| --- | --- | --- | --- | --- | --- | --- |
| 11 | Alexander | 2013 | 9 | US | Renal | 7 |
| 12 | Bosco | 2013 | 6 | Australia | Systemic | 6 |
| 13 | Buijs | 2014 | 9 | Netherlands | Other | 9 |
| 14 | Carruthers | 2014 | 72 | US | Systemic | 8* |
| 15 | Chen | 2014 | 28 | China | Systemic | 28 |
| 16 | Cheuk | 2008 | 12 | China | Lymphocutaneous | 12 |
| 17 | Detlefsen | 2012 | 63 | European | AIP/SC | 19* |
| 18 | Ebbo | 2012 | 25 | France | Systemic | 25 |
| 19 | Ghazale | 2008 | 53 | US | AIP/SC | 53 |
| 20 | Ginat | 2013 | 9 | US | Ocular | 9 |
| 21 | Go | 2012 | 14 | Korea | Ocular | 14 |
| 22 | Hart | 2012 | 978 | International | AIP/SC | 901 |
| 23 | Hirano | 2014 | 113 | Japan | Systemic | 93* |
| 24 | Huggett | 2014 | 115 | UK | AIP/SC | 115 |
| 25 | Inoue | 2011 | 17 | Japan | Other | 10 |
| 26 | Inoue | 2012 | 7 | Japan | Other | 7 |
| 27 | Ito | 2014 | 28 | Japan | Systemic | 28 |
| 28 | Kamisawa | 2011 | 58 | Japan | AIP/SC | 58 |
| 29 | Katsura | 2012 | 17 | Japan | Other | 17 |
| 30 | Kawano | 2011 | 41 | Japan | Renal | 41 |
| 31 | Khosroshahi | 2012 | 10 | US | Systemic | 10 |
| 32 | Khosroshahi | 2013 | 13 | US | Other | 13 |
| 33 | Kim | 2011 | 12 | Korea | Renal | 12 |
| 34 | Kitagawa | 2005 | 12 | Japan | Salivary | 12 |
| 35 | Kiyama | 2012 | 18 | Japan | Systemic | 18 |
| 36 | Koizumi | 2014 | 12 | Japan | Ocular | 12 |
| 37 | Kubota | 2010 | 10 | Japan | Ocular | 10 |
| 38 | Kuruma | 2013 | 78 | Japan | Systemic | 78 |
| 39 | Laco | 2013 | 5 | Czech | Other | 6 |
| 40 | Lindstrom | 2010 | 5 | US | Other | 5 |
| 41 | Masaki | 2009 | 64 | Japan | Salivary | 64 |
| 42 | Matsubayashi | 2011 | 27 | Japan | AIP/SC | 27 |
| 43 | Matsui | 2013 | 18 | Japan | Other | 18 |
| 44 | Matsuo | 2010 | 9 | Japan | Ocular | 9 |
| 45 | Mizushima | 2014 | 40 | Japan | Other | 40 |
| 46 | Moteki | 2011 | 31 | Japan | Other | 10 |
| 47 | Oh | 2010 | 16 | Japan | AIP/SC | 16 |
| 48 | Ohta | 2012 | 10 | Japan | Salivary | 10 |
| 49 | Patel | 2013 | 66 | Canada | AIP/SC | 66 |
| 50 | Raissian | 2011 | 35 | US | Renal | 27 |
| 51 | Saeki | 2013 | 43 | Japan | Renal | 43 |
| 52 | Sakamoto | 2012 | 11 | Japan | Other | 11 |
| 53 | Sato | 2008 | 21 | Japan | Ocular | 21 |
| 54 | Sato | 2012 | 40 | Japan | Lymphocutaneous | 40 |
| 55 | Sato | 2013 | 10 | Japan | Lymphocutaneous | 10 |
| 56 | Suzuki | 2012 | 23 | Japan | Systemic | 23 |
| 57 | Tabata | 2011 | 66 | Japan | Systemic | 66* |
| 58 | Takahashi | 2014 | 8 | Japan | Systemic | 8 |
| 59 | Takagi | 2013 | 8 | Japan | Salivary | 8 |
| 60 | Takagi | 2014 | 5 | Japan | Other | 5 |
| 61 | Takuma | 2010 | 56 | Japan | AIP/SC | 11 |
| 62 | Tanaka | 2014 | 43 | Japan | AIP/SC | 36 |
| 63 | Triantopoulou | 2010 | 18 | Greece | AIP/SC | 7 |
| 64 | Wallace | 2014 | 21 | US | Ocular | 21 |
| 65 | Watanabe | 2013 | 114 | Japan | Systemic | 10 |
| 66 | Wu | 2014 | 5 | Australia | Ocular | 5 |
| 67 | Yamada | 2013 | 5 | Japan | Lymphocutaneous | 5 |
| 68 | Yamamoto | 2012 | 106 | Japan | Systemic | 24 |
| 69 | Yamamoto | 2014 | 79 | Japan | Salivary | 79* |
| 70 | Yoo | 2011 | 10 | Korea | Other | 10 |
| 71 | You | 2014 | 158 | Korea | AIP/SC | 59* |
| 72 | Zen | 2013 | 19 | Japan | Other | 16 |
| *Partial information | | |  |  |  |  |

**Supplementary TABLE 3.** Failure of first-line therapies: detailed responses to rescue therapies.

| First author | Rescue therapy | Response |
| --- | --- | --- |
| Alexander | Rituximab | No |
| Alexander | Mycophenolate | Yes |
| Kubota | Glucocorticoids | Yes |
| Kubota | Glucocorticoids | Yes |
| Sato | Cyclophosphamide | Yes |
| Wallace | Methotrexate | Yes |
| Wallace | Methotrexate | No |
| Wallace | Azathioprine | No |
| Wallace | Cyclosporine A | Yes |
| Wallace | Rituximab | Yes |
| Wallace | Rituximab | Yes |
| Wallace | Rituximab | Yes |
| Wallace | Rituximab | Yes |
| Wallace | Rituximab | No |
| Wallace | Surgery | No |
| Bosco | Mycophenolate | Yes |
| Bosco | Mycophenolate | Yes |
| Cheuk | Azathioprine | No |
